# Counterfactual curiosity: motivated thinking about what might have been

**DOI:** 10.1098/rstb.2021.0340

**Published:** 2022-12-19

**Authors:** Lily Fitzgibbon, Kou Murayama

**Affiliations:** ^1^ Division of Psychology, University of Stirling, Stirling, UK; ^2^ Hector Research Institute of Education Sciences and Psychology, University of Tübingen, Tübingen, Germany; ^3^ School of Psychology and Clinical Language Sciences, University of Reading, Reading, UK; ^4^ Research Institute, Kochi University of Technology, Kochi, Japan

**Keywords:** information seeking, counterfactual, motivation, uncertainty, decision making, prediction

## Abstract

Counterfactual information, information about what might have been, forms the content of counterfactual thoughts and emotions like regret and relief. Recent research suggests that human adults and children, as well as rhesus monkeys, demonstrate ‘counterfactual curiosity’: they are motivated to seek out counterfactual information after making decisions. Based on contemporary theories of curiosity and information seeking and a broad range of empirical literature, we suggest multiple heterogeneous psychological processes that contribute to people's motivation for counterfactual information. This includes processes that are identified in the curiosity literature more generally—the potential use of counterfactual information for adaptive decision making (its long-term instrumental value) and the drive to reduce uncertainty. Additionally, we suggest that counterfactual information may be particularly alluring because of its role in causal reasoning; its relationship with prediction and decision making; and its potential to fulfil emotion regulation and self-serving goals. Some future directions have been suggested, including investigating the role of individual differences in counterfactual curiosity on learning and wellbeing.

This article is part of the theme issue ‘Thinking about possibilities: mechanisms, ontogeny, functions and phylogeny’.

## 1. Introduction

Humans are frequently pre-occupied by thinking about what might have been, imagining alternative worlds that might have transpired had past events been different. Such counterfactual worlds pervade our everyday thoughts as well as our fiction, film and even video games. For example, in Philip K. Dick's now classic novel *The Man in the High Castle* [1], an alternative world is constructed based on the change of a single past event—the successful assassination of Franklin D. Roosevelt in 1933. The story plays out in this alternative world in the 1960s in a partitioned and fascist occupied United States and has attracted a vast readership as well as a popular television adaptation. More personally, we often spend time after making decisions wondering or even seeking information about how things might have been different if we had chosen otherwise.

We refer to information relating to alternative past possibilities—i.e. what would have happened if events had been different in the past—as counterfactual information. This information forms the content of our counterfactual thoughts—thoughts about what might have been, and also counterfactual emotions such as relief and regret. There have been many studies that examined the role of counterfactual information in emotion, moral judgement, causal reasoning and decision making (e.g. [2–4]). However, much less research has paid attention to another fundamental question: what is it that makes counterfactual alternatives so captivating in the first place? To address this question, we discuss the motivational properties of counterfactual information, which we shall call *counterfactual curiosity*.

In this article, we first present evidence that human adults, children and even non-human primates are motivated to seek counterfactual information—they behaviourally exhibit counterfactual curiosity. Drawing on both empirical findings and contemporary theories of curiosity, we propose that counterfactual curiosity shares many of the same heterogeneous processes as other types of curiosity. Specifically, we suggest that it is motivated by the potential use of counterfactual information for adaptive decision making (its long-term instrumental value) and the drive to reduce uncertainty. Additionally, we suggest that counterfactual information has several inherent features that make it alluring, providing candidate processes that motivate seeking of counterfactual information. We discuss the extent to which these processes can explain seeking of counterfactual information even when it has no obvious immediate use or might elicit negative emotional experiences (such as regret). Finally, we discuss the implications of this motivational account of counterfactual information and suggest potentially fruitful avenues for future research.

## 2. Evidence for counterfactual curiosity

Counterfactual curiosity can be defined as motivation to seek out counterfactual information—information about what might have been had past events been different [5–7]. Recent research suggests that such motivation is observed after decision making in human adults and children, as well as rhesus monkeys [5–7]. There are two types of evidence that counterfactual information has a motivational lure—spontaneous counterfactual thoughts and seeking of counterfactual information.

First, humans spontaneously dedicate significant time to considering alternative possibilities, particularly after making decisions, but also when reflecting on the actions of others, and when looking back on negative life events (‘counterfactual activation’ [8]). This spontaneous generation of counterfactual information may well reflect curiosity about what might have been—we want to know, and so we dedicate time to think about the alternatives. Indeed, counterfactual thoughts pervade our everyday consciousness. For example, Markman *et al*. [9] asked participants to think aloud while playing blackjack and found that more than 90% of participants spontaneously generated counterfactual thoughts such as ‘If I had gotten the king, I would've lost to the dealer’ [9]. Further, research using an experience sampling paradigm, in which participants classified their current thoughts several times a day for 14 days, found that ‘comparison’ thoughts, including self-focused counterfactual thoughts occupy a substantial proportion of our mental landscape [10].

In addition, after traumatic life events such as the loss of a loved one, people frequently report ruminating over counterfactual thoughts. In Davis *et al*.'s [11] seminal work on this topic, subjects were interviewed several years after experiencing the loss of a spouse or child in a motor accident (study 1) and just weeks after the sudden death of an infant (study 2), and then again 18 months later. The majority of respondents (76%) reported mentally undoing the event in the weeks after an infant's death, and almost half reported still thinking ‘if only…’ thoughts years after the event. These persistent thoughts may reflect motivation for counterfactual information. While pondering this information may be disruptive and disturbing, it may nonetheless help us to make sense of negative events [12] or to make us more vigilant to future hazards. Indeed, parents who have lost a child report becoming more protective over their other children [13], although it is not known whether counterfactual thoughts play a role in this behaviour change.

Although not extensively examined, there is also evidence that children at least at the age of 8 years have spontaneous counterfactual thoughts. Guajardo *et al*. ([14], study 2) showed that, when asked to comment on events in stories, children aged between 8 and 11 years will often use counterfactual statements, particularly when the stories have unexpected or negative outcomes. Payir & Guttentag [15] demonstrated that, between the age of 8 and 12 years, children begin to use downward counterfactual statements such as ‘it could have been worse’ as a means of consoling others, but do so less than adults. Spontaneous counterfactual thinking may even emerge as early as the preschool years. Harris *et al*. ([16], experiment 3) asked 3- and 4-year olds to explain negative events and how they might have been prevented. Children referred to alternative courses of action in around a third of their explanations of the negative outcome, and in most of their suggestions for how the outcome could have been prevented. Some children (19%) spontaneously referred to alternative courses of action in their explanation of the outcome of the very first trial, prior to being prompted to think about alternative actions.

We believe that spontaneous counterfactual thought may reflect curiosity—people simulate or consider counterfactual alternatives as an epistemic exercise to gain information about what might have been. However, this interpretation of spontaneous counterfactual thinking may be subject to debate. Stronger evidence of a desire for counterfactual information comes from the actual seeking of information itself. Recent research has increasingly recognized that human adults, children and non-human primates will proactively seek out information about foregone opportunities. For example, early studies of counterfactual information seeking used vignettes and asked participants to imagine themselves in diverse scenarios—from receiving a large vet's bill for a newly acquired puppy to losing a lottery ticket—in which counterfactual information was available to them (e.g. the health of another puppy in the litter). Participants frequently reported high likelihood that they would seek information about counterfactual alternatives even when the information was likely to lead to regret [17–19]. Shani & Zeelenberg ([18], experiment 4) asked participants whether they would want to know the outcome of a lottery for which they had lost their ticket (but remembered the numbers). Participants were more likely to want to know when there was a high likelihood that they had had the winning numbers than when there was a low likelihood, suggesting that their curiosity was sufficient to overcome the potential regret of not returning the ticket.

People also seek counterfactual information after making actual decisions [20], for example, seeking information about alternative choices in gambling card games. Like counterfactual thinking more generally [21], this information seeking occurs more frequently after negative outcomes ([20], experiment 1). In a recent study, FitzGibbon *et al*. [5] presented 4- and 5-year-old children with a computerized card-matching game. After each round, children had the opportunity to use X-ray glasses to peek at the unchosen card. Most children (75%) used the glasses at least some of the time and were more likely to do so after negative outcomes (experiment 1). Interestingly, the opportunity to replay the round did not affect children's seeking of information, suggesting that they did not seek it purely for immediate instrumental gain.

Further, it has been demonstrated that people are even willing to suffer costs to seek information about foregone alternatives [6]. In an adapted Balloon Analogue Risk Task [22], participants inflated a balloon as many times as they wished, earning points with each pump, but in the knowledge that when the balloon hit a randomly determined limit, it would burst and they would win nothing. After each trial, the participants were offered the opportunity to learn where the balloon's limit was, thus learning how far they could have inflated the balloon on that trial, and how many points they could have won. This task provides an interesting test case for counterfactual curiosity because, after a win, it is likely that the information gained would lead to negative emotion (regret), and owing to the stochastic nature of the task, the information holds little instrumental value. Nonetheless, participants sought this information, even at a cost (time, physical effort and to a lesser extent money across several experiments), and that information did indeed make them feel worse. They even did so in a version of the task with only one trial—in this case, the information had no obvious and immediate value ([6], replication study). Together, these findings suggest that motivation for counterfactual information may be explained by more than its immediate instrumental value.

The desire for information about foregone alternatives is not exclusive to humans. Rhesus macaques will also seek information about foregone choice alternatives and will even suffer costs to do so [7]. In this research, macaques made choices between probabilistic gambles for water rewards. Importantly, choosing one of the gambles would additionally lead to information about the outcome of the unchosen gamble. The macaques chose the informative gamble more often than would be expected by chance, even foregoing water rewards to do so: they chose the informative gamble even when the chance of reward (expected value) was lower, suggesting that the information itself had some value. Furthermore, the macaques were sensitive to the amount of information available (the Shannon entropy of the gambles) and were more likely to forgo rewards when more information was available. This suggests that counterfactual information seeking may be analogous to other forms of information seeking in which the desire for information scales with the amount of information on offer [23].

Together, these two lines of evidence suggest that information about counterfactual alternatives holds a motivational lure. People spontaneously dedicate time to think about it and they seek information about it, even if the counterfactual information does not bring immediate benefits, and even when it comes at a cost.

## 3. The motivational underpinnings of counterfactual curiosity

The possibility of obtaining counterfactual information triggers a *motivational* state of curiosity—sometimes to the extent that people seek the information even with a lack of immediate instrumental value and at a cost. But why? Many contemporary theories of curiosity posit two related core drivers of information-seeking behaviour: potential *long-term* instrumental value (usefulness) of the information and the extent to which the information will reduce the state of uncertainty [24–26]. In the next sections, we consider the extent to which these two core drivers can explain seeking of counterfactual information.

### (a) Counterfactual information may have long-term instrumental value

Human information seeking is thought to be rational and geared towards instrumentally valuable (useful) information that serves improved decision making [27]. The instrumental value of counterfactual information is already well documented in the existing literature of counterfactual thinking. In fact, this is the core tenet of the Functional Theory of Counterfactual Thinking [28,29], that posits a specific role for counterfactual thought in enabling us to adapt our behaviour when we encounter the same, or similar, decisions in the future. After making a decision, if one recognizes that an alternative action could have led to improved performance (an upward counterfactual), then the superior action will be more likely to be selected in a future choice involving the same options. This is evidenced in both adults' and children's sequential decision making with feedback about counterfactual alternatives [30–33].

Human adults recognize this preparatory function in their everyday counterfactual thought as well as in laboratory-based studies using decision-making tasks. In a study in which participants were asked to rate negative emotions across several potential functions, people endorsed regret (the emotion associated with upward counterfactual information) higher than other negative emotions for functions including ‘Prepares me for action’ and ‘Stops me making the same mistakes again’ [34]. This suggests that some obvious instrumental value may explain some examples of counterfactual information seeking. However, we have presented several examples of counterfactual information seeking when there was no obvious and immediate instrumental value, for example, information about what might have been won in a one-shot gambling game [6]. These examples suggest that the motivation for counterfactual information goes beyond the immediate instrumental value of the information—a hallmark of curiosity [35].

So, we might ask, can potential long-term instrumental value explain counterfactual information seeking even when the information *does not* hold obvious and immediate instrumental value? Here the critical point is that, even if counterfactual information does not have an immediate instrumental value in a certain situation, there is always the possibility that the information will become useful in the future (see [36] for a similar argument). Thus, even when counterfactual information does not have obvious or immediate instrumental value, the possibility that this information might be useful in the future might nonetheless motivate its seeking.

Pushing the logic further, it is even possible to suppose that people acquire generalized belief that counterfactual information has certain instrumental value through both ontogenetic and phylogenetic processes. From childhood, we are confronted with situations in which counterfactual information is beneficial (e.g. thinking about how we could have studied more after failing an exam), so may learn to associate counterfactual information with instrumental gain. Indeed, even young children use counterfactual information to adapt their behaviour and avoid repeating errors [28,37,38]. Similarly, this generalization may occur through evolutionary processes. It is quite likely that counterfactual information improves chances of survival (e.g. by improving foraging strategies [39,40]) which may explain why even non-human animals seek counterfactual information [7]. As a result of these ontogenetic and phylogenetic processes, we tend to have strong (rather automatic) motivational reactions to counterfactual information in general, regardless of its actual instrumental value.

This kind of generalization process has been suggested as the mechanism by which reinforcement learning mechanisms can act over changing or novel stimuli and contexts—thus bringing flexibility and context independence to an otherwise rigid system [41,42]. Murayama [26] recently proposed that this generalization process has a critical role in curiosity-related information-seeking behaviour. This generalized value of information can be described as ‘diversive’ curiosity. Importantly, from this standpoint, people's behaviour of choosing to see non-instrumental counterfactual information (often with a cost) observed in past experiments (e.g. [6]) should be seen as maladaptive only in that specific experimental context—beyond the arbitrary experimental context, seeking counterfactual information is by and large beneficial to decision makers. Indeed, it is very difficult to know ahead of time what information will be valuable in the future [36].

### (b) Counterfactual information reduces uncertainty

More broadly, counterfactual information motivates behaviour because it reduces uncertainty in our representations of our actions and their consequences. In the research on curiosity, reduction of the state of uncertainty (or closing ‘information gaps’ [23,35]) has been regarded as one of the most critical sources of motivation to seek information [36,43,44]. For example, in a series of innovative laboratory studies, van Lieshout *et al*. [24] manipulated the uncertainty of the result of gambles and found that people reported that they felt more curious and were more willing to wait for information about the gamble outcome when uncertainty was high. Uncertainty has also been shown to drive everyday exploratory behaviour, for example, people's exploration of online food delivery purchases are driven by a combination of feature-based generalization of expected value and uncertainty [45]. Even infants are sensitive to uncertainty and will allocate their attention to sequences of stimuli that contain moderate levels of uncertainty [46]. Reducing uncertainty has been posited as the overarching principle guiding perception, action and learning because uncertainty reduction is critical to make good predictions about the world, helping agents to adapt to their environment [47].

This principle of uncertainty reduction identified in curiosity research should be applicable to counterfactual information. In fact, even when counterfactual information is not directly related to rewards, it still often reduces uncertainty. This is because counterfactual thought hinges on the uncertain moments in our existence, those moments where alternate events could have transpired. Shani & Zeelenberg [18] used vignettes to describe investment decisions and manipulated the level of uncertainty about the outcomes of alternative investment choices. They found that people were more likely to seek information when uncertainty was high. By considering the unknown outcomes of counterfactual alternatives, either through simulation or through information seeking, we are essentially reducing uncertainty by filling in gaps in our mental models of the relationships between our actions and their consequences. Similar arguments have recently been made for the role of uncertainty reduction in the allure of fictional worlds more generally [48] and many forms of ‘mental travel’ such as perspective taking and future thinking [49].

## 4. Motivationally relevant characteristics of counterfactual information

Thus far, the mechanisms underlying counterfactual information seeking that we have described are shared mechanisms motivating the search for other unknown information (such as the contents of an unexpected gift, or the answer to an unknown trivia question). Several contemporary models of curiosity stipulate that while curiosity is primarily triggered by expected value and uncertainty, there are several additional factors that affect the likelihood of engagement in information-seeking behaviour [23,25,26]. We argue that counterfactual curiosity has particularly strong motivational lure (in comparison to other types of curiosity) because it often involves many such characteristics that increase the attractiveness of information, which we describe below.

### (a) Counterfactual information plays a role in causal reasoning

Counterfactual information has what we consider to be fundamental use because it helps us to build *causal* models of the impact of our actions and their consequences (see [50]). Having an accurate causal model of the world is crucial to effectively make decisions to reduce uncertainty in future environments [51]. Counterfactual information plays an important role in causal reasoning and learning about causal relationships [52]. Causal representations can be defined not only by relationships between antecedents and outcomes, but also by counterfactuals—what would have happened if the antecedent had not occurred [53,54]. This is one way that causation may be distinguished from correlation—if A causes B, then changing A must lead to a change in B. Thus, consideration of counterfactual information contributes to causal reasoning, and testing counterfactual hypotheses is often part of learning about and testing causal relationships [55]. In this way, counterfactual information resolves an important type of uncertainty and helps us to build better models of the world.

Whether counterfactual information is necessary or sufficient to determine causation remains a matter of philosophical debate (see [56]), but it is clear that at the very least, humans find counterfactual information to be credible support for causal explanations. This function of counterfactual information has recently been used in the field of machine learning, where the black-box operations of deep-learning algorithms make important decisions but cannot be easily explained. Diverse sectors including healthcare and finance are increasingly reliant on machine-learning algorithms for important decision-making functions, but public trust in these decisions can be undermined by a lack of understanding of their operation. Counterfactual simulations are used to explain the behaviour of these algorithms in [57] by demonstrating what would have happened if different input values had been entered. The use of these counterfactual explanations has been found to increase understanding and trust in the decisions made by the algorithms [58].

In addition, self-relevant counterfactual information, relating to one's own decisions, can increase our sense of self-agency, giving us a further sense of causal power. In a set of novel experiments, Kulakova *et al*. [59] presented adult participants with decisions that could have identical or different outcomes to manipulate participants' sense that they could have done otherwise. Indeed, participants reported that they had more control over the outcome when the counterfactual information revealed a different result than the obtained outcome, indicating that they could have done otherwise. Conversely, adults tend to engage in counterfactual thinking more readily about events over which they had control [60]. Children are also more likely to seek counterfactual information about alternative outcomes of their own actions rather than events over which they did not have causal power ([5], experiment 2). In a card matching game, children were more likely to reveal the previously available card, and thus obtained information about what they might have won, had they chosen otherwise, over information about what was not available to them. This suggests that counterfactual curiosity may be stronger for some types of counterfactual information than others—information about controllable events (which card was selected) rather than uncontrollable events (which cards were available). This may reflect the expected instrumental value of the information, since counterfactual information about controllable events may better serve future decision making than counterfactual information about events outside of our control.

Taking the bigger picture, counterfactual thinking can also make life experiences feel more meaningful. For example, when asked to think about their decision to attend a particular college, students who were prompted to think counterfactually, by describing the ways that things could have turned out differently, felt that their college choice was more meaningful than those who were not prompted to think counterfactually [61]. Thus, counterfactual information increases our own sense of causal power and understanding of our causal role in the world.

Counterfactual information can also inform children's causal understanding, helping them to learn about and make sense of the causal order of the world. Prompting children to think counterfactually by asking them ‘what if?’ questions can facilitate their understanding of causal mechanisms. For example, prompting children to consider counterfactual alternatives can lead to controlled, disconfirmatory hypothesis testing [62], and improved evaluation of evidence when making causal judgements [63]. Consistent with our argument, researchers in developmental psychology argue that the desire to understand causal mechanisms is at the core of children's exploratory behaviour and play [64,65]. We argue that this desire is also likely to be part of children's motivation for counterfactual information. Together, this suggests that counterfactual information, particularly with that relating to our own actions, is fundamentally valuable because it helps us to better understand our causal place in the world.

### (b) Predictive processes enhance the salience of counterfactual information gaps

Information gaps about counterfactual alternatives to past choices are automatically made salient because decision making always entails some prediction. For illustration, consider choosing between two vacation destinations. You will probably make predictions about many factors such as the weather, the cost of the hotel room, the quality of the food and the other guests at both destinations, and these predictions will contribute to the decision. Predictions relating to the chosen action will be verified when the action is taken—you will know whether it is hot and sunny in your chosen holiday destination by virtue of being there. By contrast, predictions will probably remain unknown for the alternative destination unless further information is sought. This situation raises awareness of an information gap—you considered how the unchosen option would be but did not get any information about what it actually was.

In fact, Loewenstein [35] suggested that making predictions in uncertain situations increases the salience of the knowledge gap, leading to higher curiosity (i.e. motivation for information-seeking). This has recently been demonstrated experimentally: Brod & Breitwieser [66] asked participants trivia questions, and either instructed them to guess the answer, or simply told them they would see the answer. Participants who guessed the answers were more curious about the actual answer than those in the passive condition and showed a larger pupil dilation response in anticipation of the correct answer. It is worth adding that, when expectations about the chosen outcome are not met, uncertainty about the unchosen outcome may also increase, leading to increased curiosity (see [67] for evidence that the learned value of a chosen option can affect the inferred value of an unchosen option). This may explain why people tend to spontaneously engage in counterfactual reasoning after unexpected outcomes [68], and why children selectively seek counterfactual information about alternatives that they could have chosen [5].

Further evidence that predictive processes that occur during decision making can affect counterfactual judgements comes from recent findings from a study exploring adults' attention and reasoning about physical interactions between objects. Participants watched video stimuli of two moving billiard balls colliding and then rolling through a gate or failing to do so [69]. Eye movements in anticipation of balls colliding suggest that participants tracked both the actual and possible alternative trajectories of the target ball—that is where the ball actually ends up and where it would have ended up had an obstacle not been present. Furthermore, these eye movements predicted participants’ confidence in endorsing causal and counterfactual statements, suggesting that these spontaneous predictive simulations of possible trajectories played a role in causal judgement and in accumulating the content of later counterfactual thought. This hypothesized relationship between predictive processes and the content of counterfactual thought has not yet been directly tested (i.e. by manipulating whether participants are required to make explicit predictions), but could provide a fruitful avenue of future research.

### (c) Counterfactual information can serve emotion regulation and self-image preservation

People have a natural tendency to maintain positive emotions [70,71] and favourable self-identity [72,73]. Counterfactual information can play a role in maintaining positive emotions in a number of ways. For example, uncertainty itself can lead to negative emotions [74], so reducing the discomfort associated with uncertainty about foregone alternatives may motivate counterfactual information seeking. In a series of studies, Shani *et al*. [75] found that decisions to seek potentially painful information about missed opportunities were mediated by a feeling of discomfort that resulted from remaining ignorant or uncertain in both laboratory and ecologically valid settings.

In addition to reducing uncertainty about better alternatives, counterfactual information can also serve to reinforce past choices by validating good choices against worse alternatives. Counterfactual information potentially helps people enhance positive emotions because it could reveal that the agent made a good decision when compared with alternatives. In particular, people with high self-esteem tend to use downward counterfactual thoughts (those about how things could have been worse) to boost their mood when they are feeling down [76]. Thus, some people may seek counterfactual information with relentless optimism that they will learn good news that validates their choices.

Even when alternatives could have been better, counterfactual thoughts can provide excuses for poor performance—‘I would have won the race if I had better trainers' [77]. By around 8 years of age, children seem to understand this reparative function and spontaneously use counterfactual comparison to console others when they felt bad about an outcome that could have been better or worse [15], but they do not do so at adult levels until adolescence (older than 12 years). Counterfactual thinking can also serve to maintain a favourable sense of self-identity by way of self-serving or self-affirming biases. For example, Crawford & McCrea [78] presented participants with scenarios relating to polarizing public policies (gun law and capital punishment) about which their attitudes had previously been assessed. People tended to consider counterfactual alternatives that vindicated their own stance on the policy.

We have shown that counterfactual information can be emotionally arousing—it can lead to both positive emotions like relief and elation and to negative emotions like regret and disappointment. This emotional arousal in itself may be part of the appeal of counterfactual information [79]. Zuckerman & Litle [80] showed that morbid and sexual curiosity were related to the personality trait of sensation seeking. Counterfactual curiosity may also serve as a way to produce novel and arousing sensations or to alleviate boredom. In addition, engaging with emotionally arousing information may provide opportunities to challenge themselves and train their emotion regulation and stress tolerance.

## 5. Discussion

In summary, we have presented evidence that counterfactual information has a strong motivational lure and described the processes that contribute to its attractiveness. To conclude the article, we suggest several fruitful future directions to study counterfactual curiosity and better understand its role in both the activation and contents of counterfactual thought more widely.

### (a) Conflicting motives for counterfactual information

While the majority of the research we have reported points towards people desiring counterfactual information, the goals of self-preservation and emotion regulation can be in conflict with goals of reducing uncertainty. Seeking counterfactual information that helps an agent to make better decisions in future may make them feel worse in the present because they discover that another course of action would have been better. Understanding how people balance these opposing goals is important because over-valuing the self-preservation goal can lead to willful ignorance about better alternatives (i.e. ‘the ostrich problem’ [81]). In decision-making research, there are many models that attempted to address how people manage conflicting goals [70,82]. Here we have presented many candidate processes for explaining when and why counterfactual information is appealing. Future research will benefit from determining which of these processes are at work in different contexts, allowing us to better understand the relative contributions of the identified motivating factors in counterfactual information seeking. This will provide pathways to intervene in people's willful ignorance, ensuring that they engage with the information they need to make better decisions in future.

### (b) Individual differences

Although counterfactual information can hold a strong motivational lure, its seeking is not ubiquitous. The motivation for information about counterfactual alternatives is also likely to vary across individuals. The importance of individual differences has recently been recognized in the literature of curiosity (e.g. [26,83,84]), with longitudinal work demonstrating that people's information seeking may be driven by different motivations that are stable over time [83]. However, there is no research directly investigating individual differences in counterfactual curiosity, although the intra-individual variability reported in the extant research does suggest that people differ in the extent to which they seek information about foregone alternatives [6,18,19]. We suggest that there is likely to be an ideal balance in motivation for counterfactual information—too little may lead to willful ignorance and restrict people's ability to learn from mistakes, but too much may result in undue rumination and depressed mood [85,86]. For example, while the persistent counterfactual thoughts that occur after a significant loss [11] may play an important role in future decision making, they can also be extremely disruptive and disturbing. A better understanding of the motivational underpinnings of counterfactual curiosity and how this differs between individuals may provide avenues for addressing such maladaptive information-seeking strategies.

These individual differences may have developmental origins, resulting from the generalization of previous experience with counterfactual information seeking. In this regard, encouraging children to think counterfactually may be an important intervention in early education. Adults can encourage children to consider counterfactual alternatives by increasing the salience of the information gap—for example, by asking children ‘what if’ questions. If as a result of considering counterfactual alternatives, children's understanding of the world improves, then this may be the start of a positive feedback loop that increases the value of counterfactual information for young children, thus increasing the likelihood that they would seek or simulate it for themselves in the future [44,87]. Longitudinal research examining the trajectory of children's exploratory behaviour and hypothesis testing can begin to address this open question.

### (c) Ontogenetic and phylogenetic development of counterfactual curiosity

The motivational framework we have discussed suggests a mechanism by which competencies in young children and non-human animals' processing of counterfactual information may arise. Rhesus macaques and rats have been shown to be able to incorporate counterfactual information when learning the reward structure of novel environments [88,89], however, reflective cognitive processes such as backtracking and simulation of the nearest possible world, that are characteristic of mature counterfactual reasoning in human adults, are known to be challenging for young children ([90], but see [91]), and are thought to be outside the cognitive capability of non-human animals [92]. Examining how young children attend to post-decision information may give a clue as to how this may be achieved. For example, FitzGibbon *et al*. [5] found that children as young as 4 and 5 years were already motivated to learn about foregone alternatives. In a card-matching game, after making decisions and learning their outcomes, children targeted their information seeking towards cards that were genuinely available to them in the past rather than decoy cards that were not previously available. That is, they obtain the contents of counterfactuals about their own controllable events—‘If I had picked the other card…’ rather than uncontrollable events ‘If the other card had been available…’, a hallmark of adult counterfactual thinking [60].

We suggest that this targeted information seeking may be achieved by maintaining attention to information about decision alternatives (or predictions) after a decision has been made. In this way, counterfactual information may be considered and compared with factual information without the need for reflective cognitive processes such as backtracking and simulation of the nearest possible world. Instead, some instances of counterfactual judgements and comparisons may use simpler cognitive processes in some cases where motivated information seeking can provide the relevant information (see [93]). Based on the reviewed literature, we make the hypothesis that children and non-human primates will more readily seek information about alternative actions or events that they have made predictions about. This speculative hypothesis can be tested in future research using novel experimental paradigms such as the eye-tracking paradigm recently employed by Gerstenberg *et al*. [69] to elucidate the relationship between predictions and counterfactual information seeking in both human children and non-human primates. These observations suggest potential ontogenetic and phylogenetic differences in how counterfactual curiosity manifests, indicating the importance of the developmental and comparative perspectives to examine counterfactual curiosity.

## 6. Conclusion

We have demonstrated that counterfactual information is often alluring, and, by virtue of its relationship with causal reasoning and decision making, it holds particular value and salience. We have argued that both seeking and spontaneous simulation of counterfactual information result from a drive to reduce uncertainty about our own actions and our predictions about the world. By considering the motivational factors that lead to the seeking or simulation of counterfactual information, we can better understand how to use counterfactual curiosity to improve learning, decision making and wellbeing.

## Data Availability

This article has no additional data.
